# Inhibition of transforming growth factor-β signaling in myeloid cells ameliorates aortic aneurysmal formation in Marfan syndrome

**DOI:** 10.1371/journal.pone.0239908

**Published:** 2020-11-11

**Authors:** Hironori Hara, Sonoko Maemura, Takayuki Fujiwara, Norifumi Takeda, Satoshi Ishii, Hiroki Yagi, Takaaki Suzuki, Mutsuo Harada, Haruhiro Toko, Tsubasa Kanaya, Hideaki Ijichi, Harold L. Moses, Eiki Takimoto, Hiroyuki Morita, Hiroshi Akazawa, Issei Komuro

**Affiliations:** 1 Department of Cardiovascular Medicine, The University of Tokyo Hospital, Bunkyo-ku, Tokyo, Japan; 2 Department of Advanced Translational Research and Medicine in Management of Pulmonary Hypertension, The University of Tokyo Hospital, Bunkyo-ku, Tokyo, Japan; 3 Department of Gastroenterology, The University of Tokyo Hospital, Bunkyo-ku, Tokyo, Japan; 4 Vanderbilt-Ingram Comprehensive Cancer Center, Vanderbilt University, Nashville, Tennessee, United States of America; Niigata Daigaku, JAPAN

## Abstract

Increased transforming growth factor-β (TGF-β) signaling contributes to the pathophysiology of aortic aneurysm in Marfan syndrome (MFS). Recent reports indicate that a small but significant number of inflammatory cells are infiltrated into the aortic media and adventitia in MFS. However, little is known about the contribution of myeloid cells to aortic aneurysmal formation. In this study, we ablated the TGF-β type II receptor gene *Tgfbr2* in myeloid cells of *Fbn1*^*C1039G/+*^ MFS mice (*Fbn1*^*C1039G/+*^;*LysM-Cre/+*;*Tgfbr2*^*fl/fl*^ mice, hereinafter called *Fbn1*^*C1039G/+*^;*Tgfbr2*^*MyeKO*^) and evaluated macrophage infiltration and TGF-β signaling in the aorta. Aneurysmal formation with fragmentation and disarray of medial elastic fibers observed in MFS mice was significantly ameliorated in *Fbn1*^*C1039G/+*^;*Tgfbr2*^*MyeKO*^ mice. In the aorta of *Fbn1*^*C1039G/+*^;*Tgfbr2*^*MyeKO*^ mice, both canonical and noncanonical TGF-β signals were attenuated and the number of infiltrated F4/80-positive macrophages was significantly reduced. *In vitro*, TGF-β enhanced the migration capacity of RAW264.7 macrophages. These findings suggest that TGF-β signaling in myeloid cells promotes aortic aneurysmal formation and its inhibition might be a novel therapeutic target in MFS.

## Introduction

MFS is a systemic disorder of connective tissues caused by pathogenic variants in the *FBN1* gene, which encodes the major component of the extracellular matrix microfibril fibrillin-1 [1]. Fibrillin-1 binds to the latent complex of TGF-β and regulates its activity in response to pathophysiological stresses. *FBN1* pathogenic variants that affect the structure or function of fibrillin-1 protein not only cause structural weakness of the connective tissue but also stimulate improper latent TGF-β activation [2, 3]. Among the diverse manifestations of MFS, aortic aneurysm and dissection are the most serious and the leading causes of death [4]. The aortic TGF-β signaling is activated in MFS, and treatment of adult MFS mice with a TGF-β neutralizing antibody (TGF-NAb) prevents aortic root dilatation associated with attenuation of the canonical Smad and the noncanonical extracellular signal-regulated kinase (ERK) signaling pathways [5]. On the contrary, early initiation of TGF-NAb treatment from postnatal day 16 (P16) in fibrillin-1 hypomorphic (*Fbn1*^*mgR/mgR*^) mice exacerbates aneurysmal formation, but the initiation from P45 ameliorates it [6]. Adult mice with a conditional tamoxifen-inducible biallelic disruption of *Tgfbr2* in smooth muscle cells (*Myh11-CreER*^*T2*^;*Tgfbr2*^*fl/fl*^) develop aortic aneurysm [7]. These findings suggest that TGF-β signaling plays pivotal roles both in early postnatal aortic development and adult aortic homeostasis.

Recent studies show that a small but significant number of inflammatory cells are infiltrated into the aortic media and adventitia [8, 9]. Importantly, the number of inflammatory cells infiltrated into the aorta was larger in patients who required prophylactic repair of the aorta early in life, compared to patients who did not need prophylactic repair until elderly [8]. It is possible that inflammatory cells play a crucial role in aortic aneurysmal formation.

Here, we hypothesized that upregulated TGF-β activity in myeloid cells plays an important role in the disease progression. In the present study, we ablated the *Tgfbr2* gene in myeloid cells of *Fbn1*^*C1039G/+*^ MFS mice and evaluated the effect on aortic aneurysmal formation in MFS.

## Methods

### Animals

Generation of homozygous *Tgfbr2*-floxed (*Tgfbr2*^*fl/fl*^) mice and genotyping have been previously described [10]. *Fbn1*^*C1039G/+*^ and *LysM-Cre/+* mice were obtained from the Jackson Laboratory [11, 12]. All animal experiments were approved by the University of Tokyo Ethics Committee for Animal Experiments and strictly adhered to the guidelines of the University of Tokyo for animal experiments. Mice were monitored daily by animal facility staff and observed for signs of stress and distress.

### Efficacy of Cre-mediated recombination in *Tgfbr2*^*MyeKO*^ mice

To confirm the previously reported high-efficacy of Cre-mediated recombination in *Tgfbr2*^*MyeKO*^ mice [13], macrophages were obtained from the peritoneal cavity of 6-week-old mice 4 days after injection of 4% thioglycollate (211260; Becton Dickinson and Company). Total RNA isolation, cDNA conversion, and semi-quantitative reverse transcription polymerase chain reaction (PCR) were performed as previously reported [14, 15]. The PCR primers used were as follows (forward and reverse, respectively): *Tgfbr2*, 5’-GCAAGTTTTGCGATGTGAGA-3’ and 5’-GGCATCTTCCAGAGTGAAGC-3’; *18S* rRNA, 5’-CGGCTACCACATCCAAGGAA-3’, and 5’-AGCTGGAATTACCGCGGC-3’.

### Noninvasive blood pressure measurement and ultrasonography

Systolic blood pressure was measured using a tail-cuff method (MK-2000ST; Muromachi Kikai). Ultrasonography was performed using the Vevo 2100 Imaging System (VisualSonics) while mice were anesthetized with 2.5% isoflurane. The luminal diameters of the aortic root were measured at the age of 8, 12, and 30 weeks by B-mode imaging.

### Macroscopic images and histological analysis

Before excision of aorta, mice were euthanized by isoflurane overexposure. Following intra-cardiac perfusion of 4% paraformaldehyde, thoracic aorta was cleared of surrounding tissue and digitally captured using Leica MZ10 F microscope and Leica DFC450 C camera.

Tissue sections (5 μm) were prepared at the proximal ascending aorta (just above sinotubular junction). Elastica van Gieson staining was used to visualize the aortic elastic fiber structure. Elastic fiber breaks were defined as the presence of discernable breaks of continuous elastin fiber and counted circumferentially in all laminae [16, 17]. Rat anti-mouse monoclonal F4/80 antibody (MCA497; Bio-Rad) was used for immunohistochemical staining to recognize macrophages at the dilution of 1:200. The VECTASTAIN Elite ABC system (PK-6104; Vector Laboratories) was used for sensitive avidin/biotin-based peroxidase system and hematoxylin was used for counterstain of nuclei. The number of infiltrated macrophages was determined by counting cells at 40× magnification from four random fields, and the average number per area of aortic adventitia was used as an individual value [18, 19]. Rabbit-anti-mouse monoclonal phospho-Smad2/3 antibody (#3108; Cell Signaling Technology) was used as a primary antibody at the dilution of 1:100. The signal was visualized by ImmPRESS Reagent (MP-7401; Vector Laboratories) and TSA Fluorescein (SAT701B001EA; Perkin Elmer). DAPI (D523; Wako) was used for nuclei staining prior to mounting with VECTASHIELD (H-1000; Vector Laboratories). The ratio of positively stained nuclei to the total number of nuclei in the aorta was determined at 20× magnification from three random fields, and the average percentage was used as an individual value [20, 21]. Rabbit-anti-mouse polyclonal phospho-ERK1/2 antibody (#9101; Cell Signaling Technology) was used as a primary antibody at the dilution of 1:200. The signal was visualized by ImmPRESS Reagent (MP-7401; Vector Laboratories). 3, 3'-Diaminobenzidine tetrahydrochloride (SK-4100; Vector Laboratories) was used as substrate for horseradish peroxidase. The ratio of positively stained area to the total area of the aorta was determined at 40× magnification from three random fields, and the average percentage was used as an individual value [22]. Digital images of tissue sections were obtained using Olympus BX 51 microscope and Olympus DP 70 camera. Brightness and contrast were adjusted linearly across the entirety of each image.

### MTS cell proliferation assay

RAW264.7 cells under fetal calf serum-free starved condition were incubated with the vehicle or 5 ng/mL of recombinant human TGF-β1 (209–16544; Wako) for 24 hours. Then, the cell proliferation rate was evaluated using a CellTiter 96® AQueous One Solution Cell Proliferation Assay kit (G3582; Promega) and a microplate-reading luminometer (Wallac Arvo Mx 1420; PerkinElmer) [23].

### *In vitro* cell migration assay

RAW264.7 cells were grown to confluence before the cell monolayer was scratched using a sterile P200 micropipette tip [24]. Next, cells were washed with phosphate-buffered saline, cultured at 37°C in fetal calf serum-free DMEM and treated with the vehicle or 5 ng/mL of recombinant human TGF-β1 (Wako) for 24 hours. The images of the scratched area were captured immediately after the scratch and after 24 hours to monitor cell migration. The migration capacity was quantified from the area between the edges of cells in the scratch zone.

### Statistical analysis

All analyses were performed using JMP Pro 14.2.0 (SAS Institute). Data are shown as mean ± standard deviation. The assumption of homogeneity of variance was tested using Levene’s test. The significance of differences among means was evaluated using Student’s *t*-test, Welch’s *t*-test, one-way analysis of variance followed by post hoc Tukey–Kramer test, or Kruskal–Wallis test followed by post hoc Steel–Dwass test. *P*-values < 0.05 were considered statistically significant.

## Results

To examine the impact of TGF-β signaling in myeloid cells on aortic aneurysmal formation in MFS, we ablated *Tgfbr2* in myeloid cells in *Fbn1*^*C1039G/+*^ heterozygous MFS mice by crossing with myeloid-specific *Tgfbr2* knockout mice (*Tgfbr2*^*MyeKO*^ mice). Exon 2 of *Tgfbr2* (169 bp) was flanked with loxP sites and Cre-mediated recombination produced out-of-frame transcripts in myeloid cells of *Tgfbr2*^*MyeKO*^ mice (S1 Fig) [10, 13]. Compared to control wild-type mice (*Tgfbr2*^*fl/fl*^) and MFS mice (*Fbn1*^*C1039G/+*^;*Tgfbr2*^*fl/fl*^), myeloid-specific *Tgfbr2* disruption did not affect systolic blood pressure or body weight of *Tgfbr2*^*MyeKO*^ and *Fbn1*^*C1039G/+*^;*Tgfbr2*^*MyeKO*^ mice (S2 Fig).

**Fig 1:**
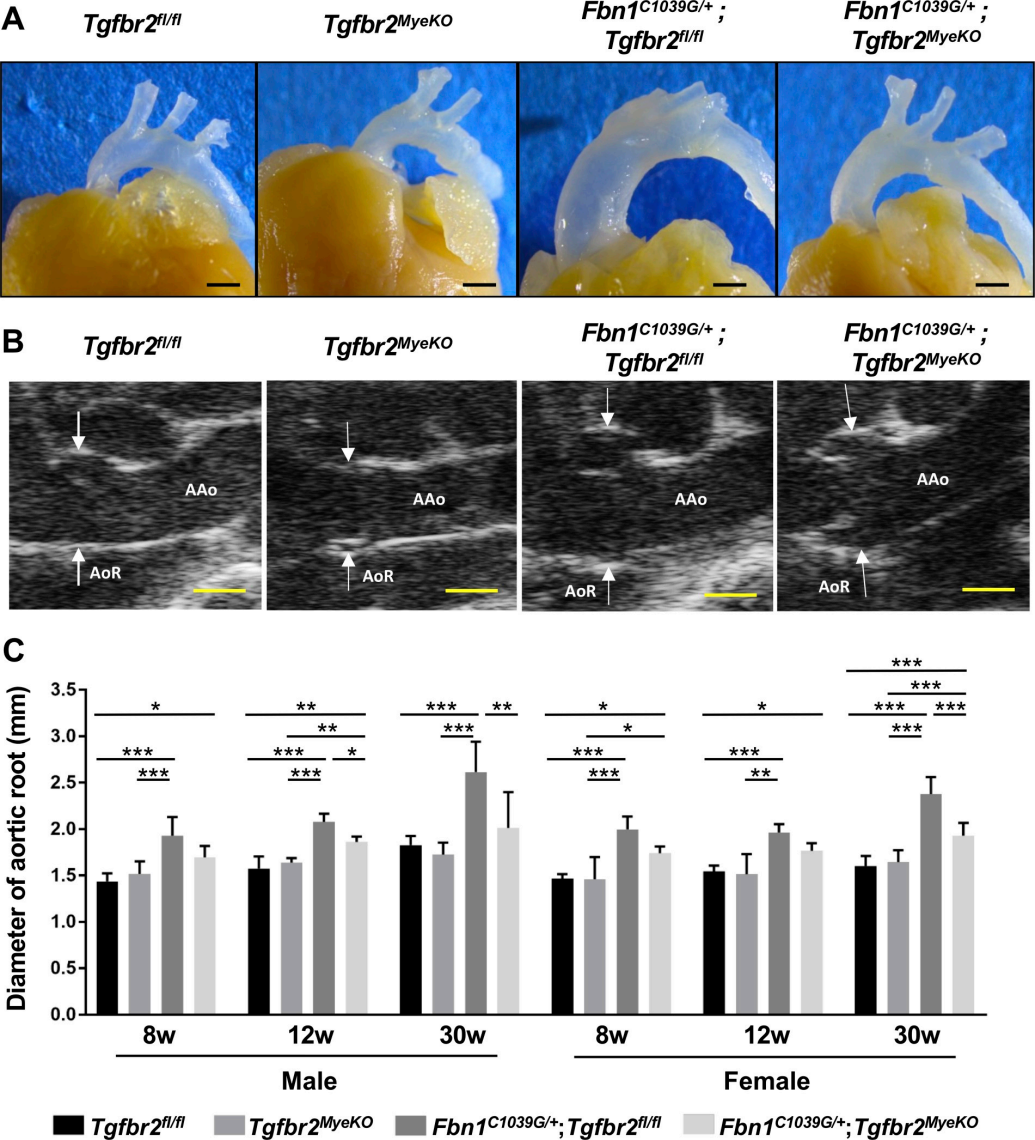
Myeloid-specific ablation of *Tgfbr2* ameliorated aortic aneurysm in *Fbn1*^*C1039G/+*^ mice. (A, B) Representative macroscopic appearance (A) and ultrasound images (B) of the thoracic aorta at 30 weeks of age in *Tgfbr2* ^*fl/fl*^, *Tgfbr2*^*MyeKO*^, *Fbn1*^*C1039G/+*^;*Tgfbr2* ^*fl/fl*^, and *Fbn1* ^*C1039G/+*^;*Tgfbr2*^*MyeKO*^ mice. Scale bar, 1 mm. Arrows indicate aortic root (AoR); AAo, Ascending aorta. (C) Aortic root diameter measured by ultrasonography at 8, 12, and 30 weeks of age. n = 3–12 per group. Data are shown as mean ± standard deviation. *p < 0.05, **p < 0.01, ***p < 0.001.

**Fig 2:**
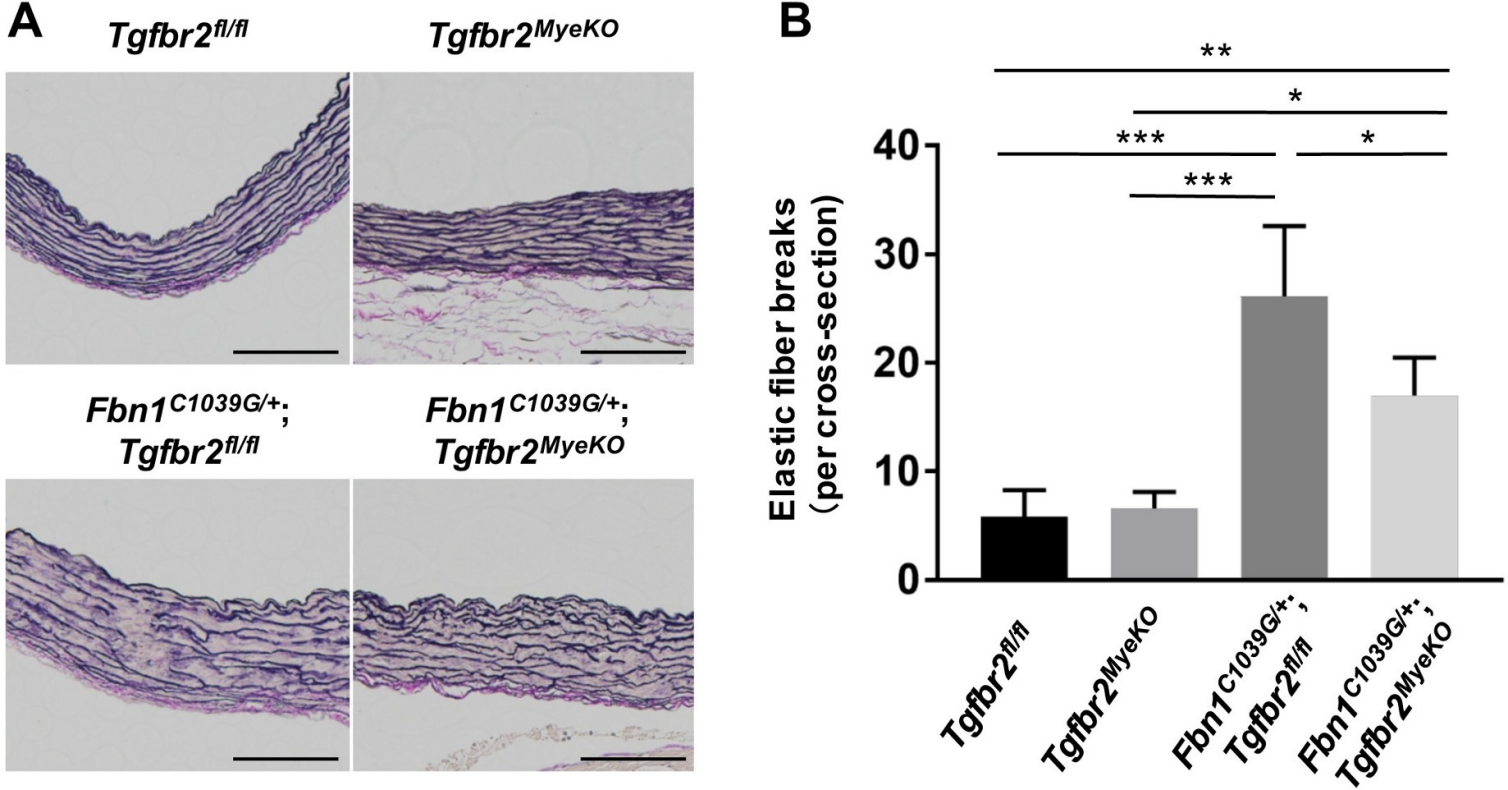
Myeloid-specific ablation of *Tgfbr2* reduced aortic medial thickening and elastic fiber fragmentation in *Fbn1*^*C1039G/+*^ mice. (A) Representative images of Elastica van Gieson staining of the proximal ascending aorta at 30 weeks of age in *Tgfbr2* ^*fl/fl*^, *Tgfbr2*^*MyeKO*^, *Fbn1*^*C1039G/+*^;*Tgfbr2* ^*fl/fl*^, and *Fbn1* ^*C1039G/+*^;*Tgfbr2*^*MyeKO*^ mice. Scale bar, 100 μm. (B) Average number of elastic fiber breaks per section. All mice were male. n = 4–7 per group. Data are shown as mean ± standard deviation. *p < 0.05, **p < 0.01, ***p < 0.001.

*Fbn1*^*C1039G/+*^;*Tgfbr2*^*fl/fl*^ MFS mice developed progressive aortic root dilatation to 30 weeks of age without early death. On the other hand, aneurysmal formation was significantly suppressed in both male and female *Fbn1*^*C1039G/+*^;*Tgfbr2*^*MyeKO*^ mice, and *Tgfbr2*^*MyeKO*^ mice did not develop any aortic phenotypes (Fig 1). Histological analysis revealed aberrant thickening of the aortic media with fragmentation and disarray of elastic fibers in *Fbn1*^*C1039G/+*^;*Tgfbr2*^*fl/fl*^ mice, but they were markedly reduced in *Fbn1*^*C1039G/+*^;*Tgfbr2*^*MyeKO*^ mice (Fig 2). These findings suggest that TGF-β signaling in myeloid cells is inessential in normal aortic development and homeostasis but it promotes the progression of aortic aneurysmal formation in MFS.

**Fig 3:**
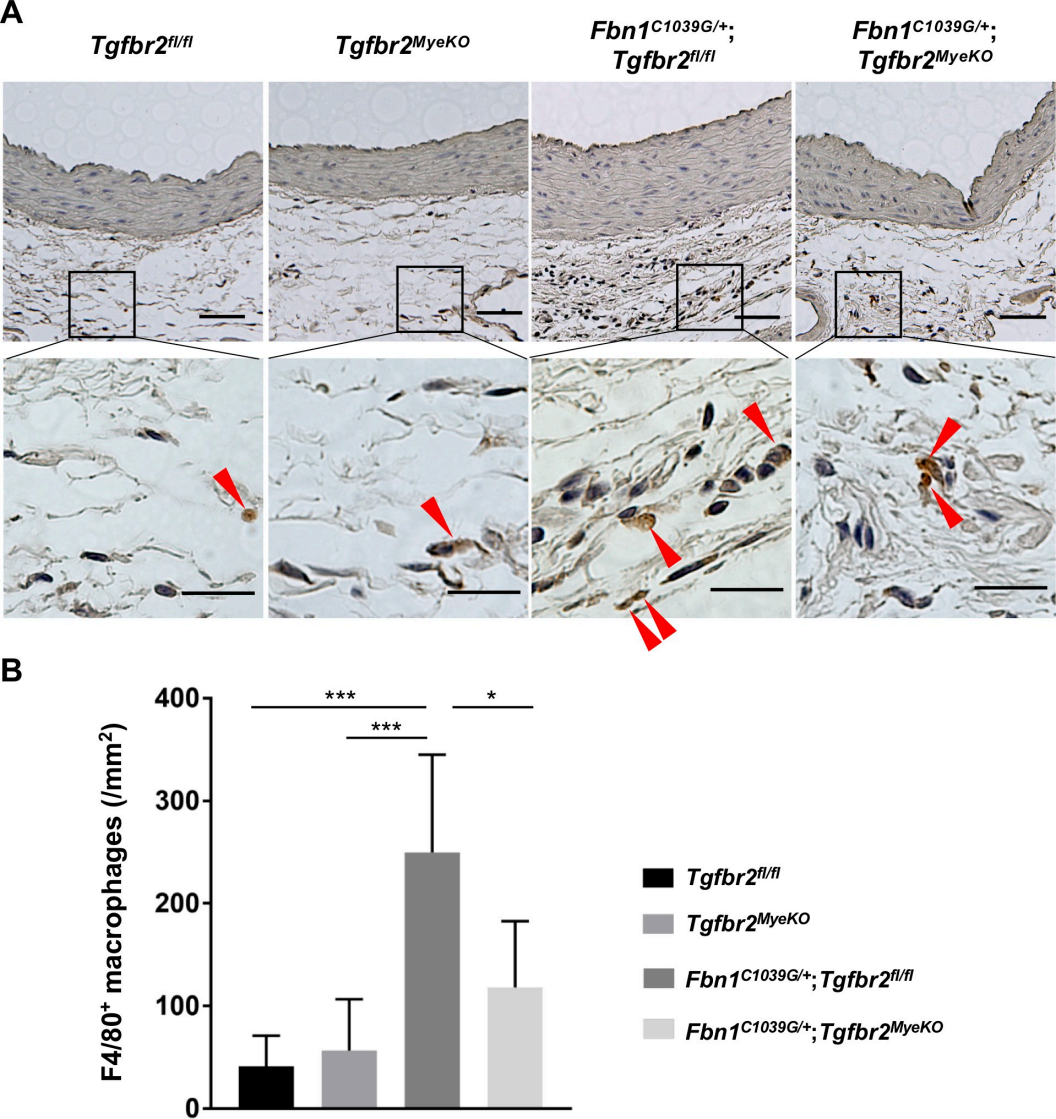
Myeloid-specific ablation of *Tgfbr2* reduced macrophage infiltration into the aortic wall in *Fbn1*^*C1039G/+*^ mice. (A) Representative images of F4/80 (marker of macrophage, red arrowheads) of the proximal ascending aorta at 30 weeks of age in *Tgfbr2* ^*fl/fl*^, *Tgfbr2*^*MyeKO*^, *Fbn1*^*C1039G/+*^;*Tgfbr2* ^*fl/fl*^, and *Fbn1* ^*C1039G/+*^;*Tgfbr2*^*MyeKO*^ mice. Cell nuclei were counterstained with hematoxylin. All mice were male. Scale bar, 50 μm (20 μm in inset). (B) Average number of F4/80-positive macrophages per area of aortic adventitia. Four random 40× magnification fields were investigated. All mice were male. n = 4–7 per group. Data are shown as mean ± standard deviation. *p < 0.05, ***p < 0.001.

Next, we evaluated macrophage infiltration into the aneurysmal wall, as macrophage is reported to be a major component of infiltrating inflammatory cells [8]. F4/80-positive macrophages were significantly increased mainly in the adventitia of the proximal ascending aorta (just above sinotubular junction) of *Fbn1*^*C1039G/+*^;*Tgfbr2*^*fl/fl*^ mice at 30 weeks of age. However, the number of macrophages infiltrated into the aorta of *Fbn1*^*C1039G/+*^;*Tgfbr2*^*MyeKO*^ mice was significantly reduced compared to *Fbn1*^*C1039G/+*^;*Tgfbr2*^*fl/fl*^ mice (Fig 3).

**Fig 4:**
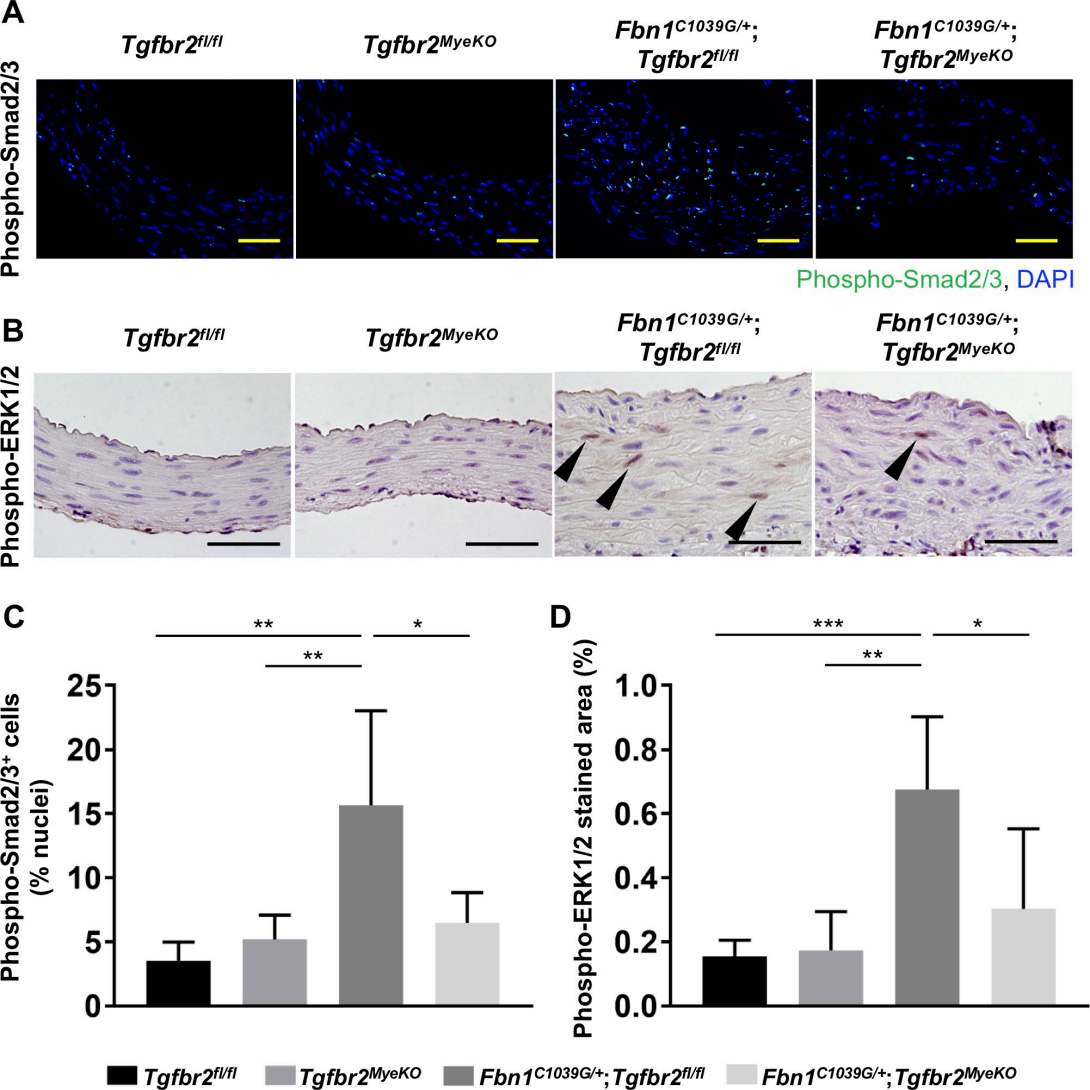
Myeloid-specific ablation of *Tgfbr2* attenuated phospho-Smad2/3 and phospho-ERK1/2 signals in *Fbn1*^*C1039G/+*^ mice. (A, B) Representative phospho-Smad2/3 (A) and phospho-ERK1/2 (B) staining of the proximal ascending aorta at 30 weeks of age in *Tgfbr2* ^*fl/fl*^, *Tgfbr2*^*MyeKO*^, *Fbn1*^*C1039G/+*^;*Tgfbr2* ^*fl/fl*^, and *Fbn1* ^*C1039G/+*^;*Tgfbr2*^*MyeKO*^ mice. Scale bar, 50 μm. Arrowheads show the representative positive staining. (C) Average percentage of positive staining of phospho-Smad2/3 per nuclei. Three random 20× magnification fields were investigated. All mice were male. n = 4–7 per group. Data are shown as mean ± standard deviation. *p < 0.05, **p < 0.01. (D) Average percentage of positive stained area of phospho-ERK1/2 per aortic area. Three random 40× magnification fields were investigated. All mice were male. n = 4–7 per group. Data are shown as mean ± standard deviation. *p < 0.05, **p < 0.01, ***p < 0.001.

We further examined the effect of myeloid-specific *Tgfbr2* disruption on TGF-β signaling-related pathways in the aortic wall. Increased Smad2/3 and ERK1/2 phosphorylations were evident in the ascending aorta of *Fbn1*^*C1039G/+*^;*Tgfbr2*^*fl/fl*^ mice, but they were remarkably attenuated in *Fbn1*^*C1039G/+*^;*Tgfbr2*^*MyeKO*^ mice (Fig 4).

**Fig 5:**
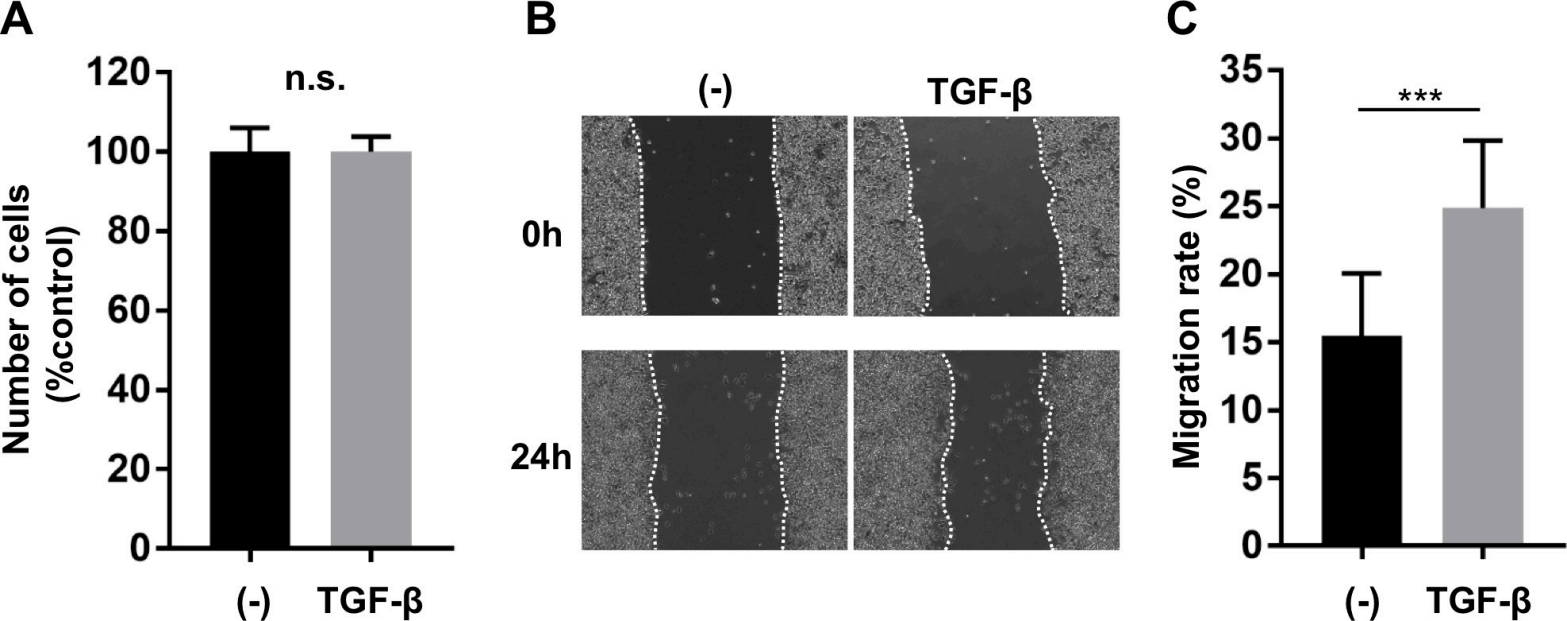
Effects of TGF-β treatment on the proliferation and migration capacity of RAW264.7 macrophages. (A) MTS cell proliferation assay. n = 6 per group. Data are shown as mean ± standard deviation. n.s, not significant. (B, C) *In vitro* cell migration assay. A monolayer of RAW264.7 cells was scratched with a P200 pipette tip, which induced migration of cells into the denuded area to close the wound in the presence or absence of TGF-β (5 ng/mL) for 24 hours. (B) Representative images obtained immediately after the scratch and after 24 hours. (C) The migration capacity was quantified from the area between the edges of cells in the scratch zone. n = 10 per group. Data are shown as mean ± standard deviation. ***p < 0.001.

Finally, we investigated the effect of TGF-β on the migration capacity of macrophage using an *in vitro* monolayer wound healing assay. After RAW264.7 cells, a murine macrophage cell line, reached confluence, an artificial gap was created in the macrophage monolayer using a straight line. TGF-β treatment (5 ng/mL) did not promote cell proliferation (Fig 5A) but significantly enhanced the wound closure rate 24 hours after scratching (Fig 5B and 5C). These data indicates that TGF-β could upregulate the migration capacity of RAW264.7 macrophages.

## Discussion

In this study, we examined whether TGF-β signaling in myeloid cells plays a crucial role in aortic aneurysmal formation. Myeloid-specific *Tgfbr2* gene ablation in MFS mice attenuated activation of TGF-β signaling and macrophage infiltration in the aorta. In *Fbn1* ^*C1039G/+*^;*Tgfbr2*^*MyeKO*^ mice, aortic dilatation was already evident at 8 and 12 weeks of age, but it became less prominent as they grew up (Fig 1C). Therefore, myeloid-specific deletion of *Tgfbr2* in MFS model mice ameliorated aortic aneurysmal formation, but it did not prevent the initiation of aortic aneurysmal formation. In addition, TGF-β enhanced the migration capacity of RAW264.7 macrophages *in vitro*. These findings suggest that TGF-β signaling in myeloid cells might promote the progression of aortic aneurysmal formation, and its inhibition might be a novel therapeutic target in MFS.

*Tgfbr2*^*MyeKO*^ mice, lacking myeloid-specific TGF-β receptor II, but with normal hematopoiesis, have been used to investigate disease-specific roles of TGF-β signaling in myeloid cells [25–28]. In the present study, *Tgfbr2* disruption in myeloid cells attenuated phosphorylations of Smad2/3 and ERK1/2 in the ascending aorta of MFS mice (Fig 4). In MFS, mutated *FBN1* gene causes reduced or abnormal fibrillin-1 protein, which normally regulates TGF-β bioavailability by releasing latent TGF-β from the extracellular matrix in response to pathophysiological stimuli. Thus, the ensuing upregulation of TGF-β signaling cascades play crucial roles in MFS [3]. Angiotensin II type I blocker losartan has been reported to remarkably ameliorate activation of Smad2 and aortic aneurysmal formation in *Fbn1*^*C1039G/+*^ mice [29], but the mechanism is still not fully understood. It has also been demonstrated that the activation of ERK1/2 as the noncanonical pathway of TGF-β signaling serves as a prominent driver of aortic aneurysmal formation and ERK1/2 signal is inhibited by systemic TGF-β-neutralizing antibody treatment in *Fbn1*^*C1039G/+*^ mice [5]. Furthermore, selective inhibition of ERK1/2 activation ameliorated aortic growth in *Fbn1*^*C1039G/+*^ mice, whereas Smad2 signal was unchanged [5]. These data suggest that TGF-β-driven ERK1/2 activation strongly contributes to aneurysmal formation in MFS, and thus further investigation is needed to elucidate the role of TGF-β signaling in myeloid cells in the ERK1/2 phosphorylation in the aortic wall.

It has been reported that pre-treatment of TGF-β exerts biphasic effect on the migration capacity of RAW264.7 cells [30]. However, in the present study, continuous stimulation of TGF-β for 24 hours enhanced the migration capacity of RAW264.7 macrophages (Fig 5). We also demonstrated that *Tgfbr2* disruption in myeloid cells reduced macrophage infiltration into the aortic wall (Fig 3). One possible reason for amelioration of aortic aneurysmal formation by myeloid-specific disruption of *Tgfbr2* in MFS is the inhibitory effect on macrophage migration and invasion into the diseased aortic wall. Myeloid-specific deletion of *Tgfbr2* is reported to reduce migration and invasion capacity of macrophages in tumor-bearing mice as well [26]. Myeloid-specific deletion of *Tgfbr2* might alter the expression of adhesion molecules and cytokines and modulate migration capacity. Co-culture of *FBN1*-knocked-down vascular smooth muscle cells and macrophages derived from *Tgfbr2*^*MyeKO*^ mice could partially recapitulate the *in vivo* context of MFS.

It has been shown that IL-6 recruits CCR2-positive monocytes which serve to amplify the inflammatory cascade, and IL-6 signaling contributes to Angiotensin II-induced aortic aneurysm [31]. Activation of IL-6 signaling has been demonstrated in hypomorphic *Fbn1*^*mgR/mgR*^ mice [32]. Additionally, in the aorta of MFS and familial thoracic aortic aneurysm and dissection, the expression of adhesion molecules surrounding the vasa vasorum in the adventitia is increased [8]. Taken together, reduction of macrophage invasion into the diseased aortic wall might suppress IL-6 signaling and adhesion molecules in the aneurysmal wall of *Fbn1*^*C1039G/+*^;*Tgfbr2*^*MyeKO*^ mice. This hypothesis should be confirmed in the near future.

TGF-β signaling has been shown to play an important role in phenotypic differentiation of macrophage in various pathological situations [33, 34]. Hematopoietic-specific *Tgfbr2* deletion has been reported to suppress the expression of Galectin-3, which regulates M2 macrophage polarization, in bone marrow-derived macrophage/monocytes [33]. TGF-β inhibits M1 polarization and promotes M2 polarization in human THP-1 macrophage [34]. Although, to the best of our knowledge, the polarization of macrophage in MFS has not been evaluated, we speculate that phenotypic alteration of macrophage as well as change in the migration capacity due to increased TGF-β signaling may contribute to the progression of aortic aneurysm in MFS. Further investigation of M1/M2 phenotypic alteration and migration capacity of bone marrow-derived macrophage/monocytes from *Fbn1*^*C1039G/+*^;*Tgfbr2*^*MyeKO*^ mice is needed to elucidate the mechanism.

This study demonstrated for the first time that TGF-β signaling disruption in myeloid cells ameliorated *Fbn1*^*C1039G/+*^ MFS aortopathy, where F4/80-positive macrophage infiltration and both canonical and noncanonical TGF-β signals were markedly reduced. Our findings suggest that inhibition of TGF-β signaling in myeloid cells might be a novel therapeutic target in MFS.

## Supporting information

S1 Checklist The ARRIVE guidelines checklist. (PDF)

S1 Fig Efficacy of Cre-mediated loxP recombination in myeloid cells. Semi-quantitative PCR of cDNA prepared from peritoneal macrophages was performed. PCR products for *Tgfbr2* and *18s* are 197 bp and 188 bp.
(TIF)

S2 Fig Effects of *Tgfbr2* deletion in myeloid cells on blood pressure and body weight. Systolic blood pressure (A) and body weight (B) at 30 weeks of age in *Tgfbr2* ^*fl/f*^, *Tgfbr2*^*MyeKO*^, *Fbn1*^*C1039G/+*^;*Tgfbr2* ^*fl/fl*^, and *Fbn1* ^*C1039G/+*^;*Tgfbr2*^*MyeKO*^ mice. n = 4–12 per group. Data are shown as mean ± standard deviation. n.s, not significant.
(TIF)

S1 Raw image Original image of gel (for S1 Fig). Products amplified by semi-quantitative reverse transcription polymerase chain reaction using *Tgfbr2* and *18s* primers were separated by 1.2% agarose gel electrophoresis.
(PDF)
