# Supplementary figures and images for: Inhibition of transforming growth factor-β signaling in myeloid cells ameliorates aortic aneurysmal formation in Marfan syndrome
Source: PLoS One. 2020 Nov 11;15(11):e0239908. doi: 10.1371/journal.pone.0239908 (PMC7657512; doi:10.1371/journal.pone.0239908)

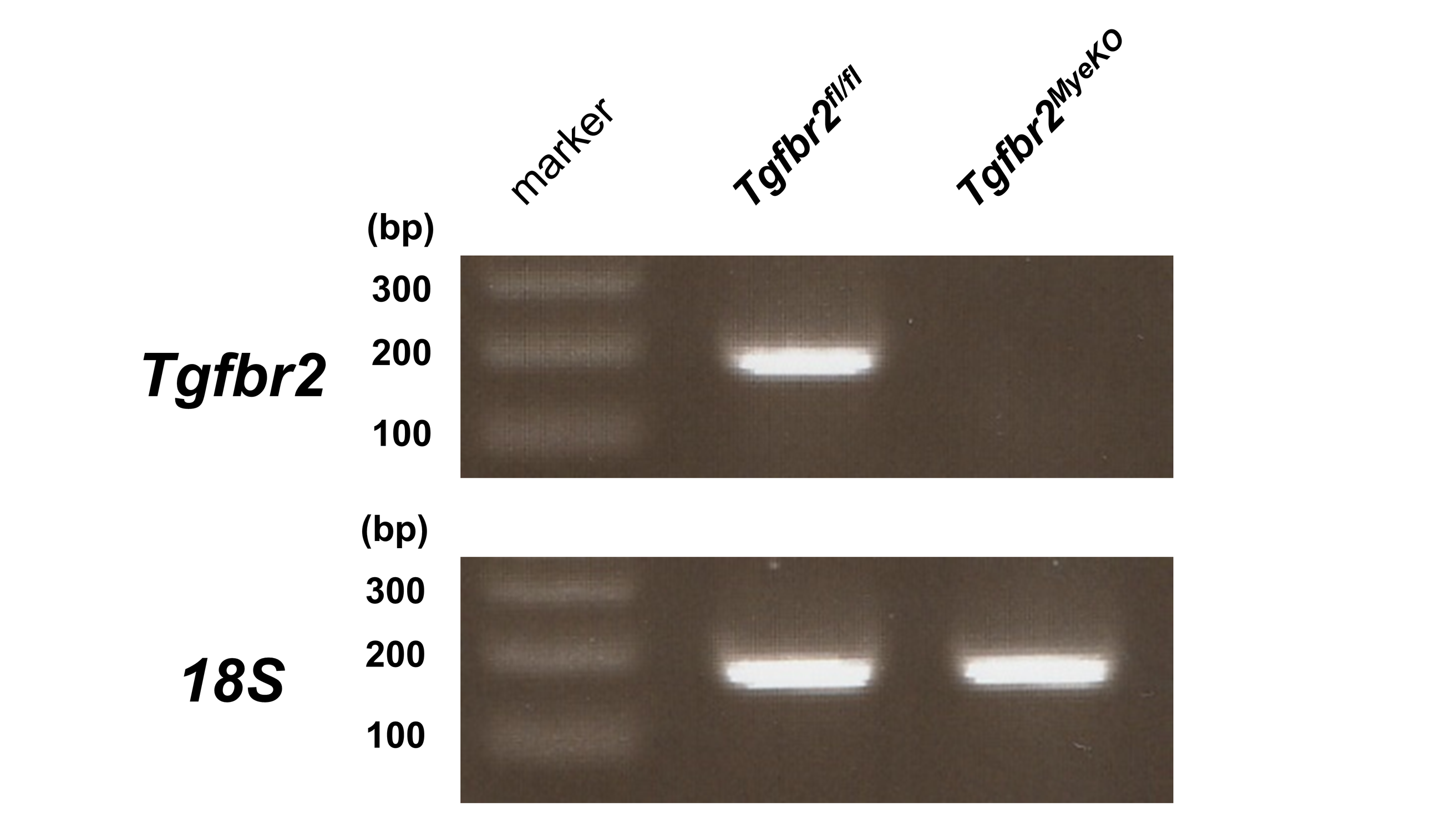

Supplement: S1 Fig — Semi-quantitative PCR of cDNA prepared from peritoneal macrophages was performed. PCR products for Tgfbr2 and 18s are 197 bp and 188 bp. (TIF) [file pone.0239908.s002.tif]

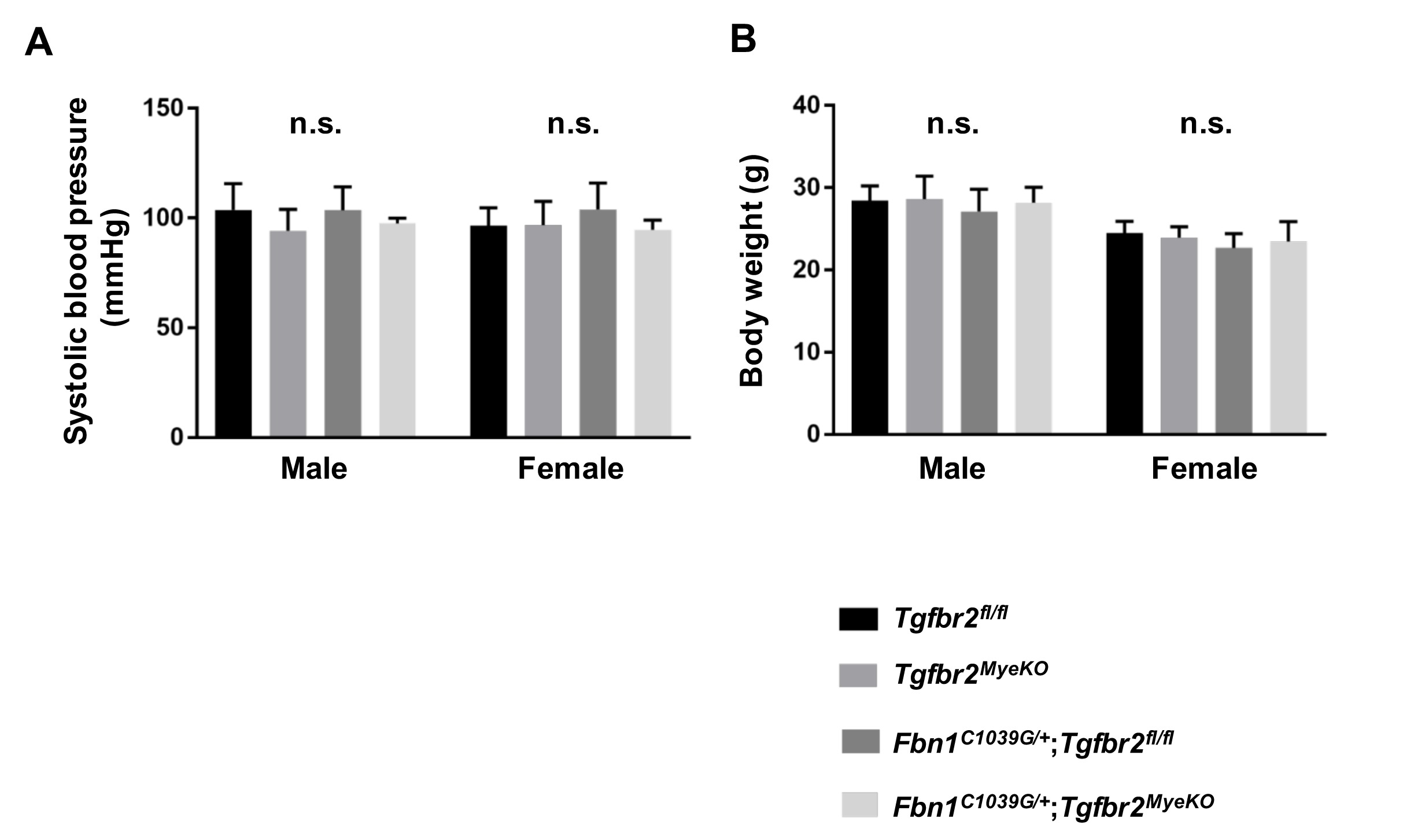

Supplement: S2 Fig — Systolic blood pressure (A) and body weight (B) at 30 weeks of age in Tgfbr2 fl/f, Tgfbr2MyeKO, Fbn1C1039G/+;Tgfbr2 fl/fl, and Fbn1 C1039G/+;Tgfbr2MyeKO mice. n = 4–12 per group. Data are shown as mean ± standard deviation. n.s, not significant. (TIF) [file pone.0239908.s003.tif]
