# Supplementary material for: Inhibition of transforming growth factor-β signaling in myeloid cells ameliorates aortic aneurysmal formation in Marfan syndrome
Source: PLoS One. 2020 Nov 11;15(11):e0239908. doi: 10.1371/journal.pone.0239908 (PMC7657512; doi:10.1371/journal.pone.0239908)
Supplement: S1 Raw image — Products amplified by semi-quantitative reverse transcription polymerase chain reaction using Tgfbr2 and 18s primers were separated by 1.2% agarose gel electrophoresis. (PDF) [file pone.0239908.s004.pdf]

S1 Fig

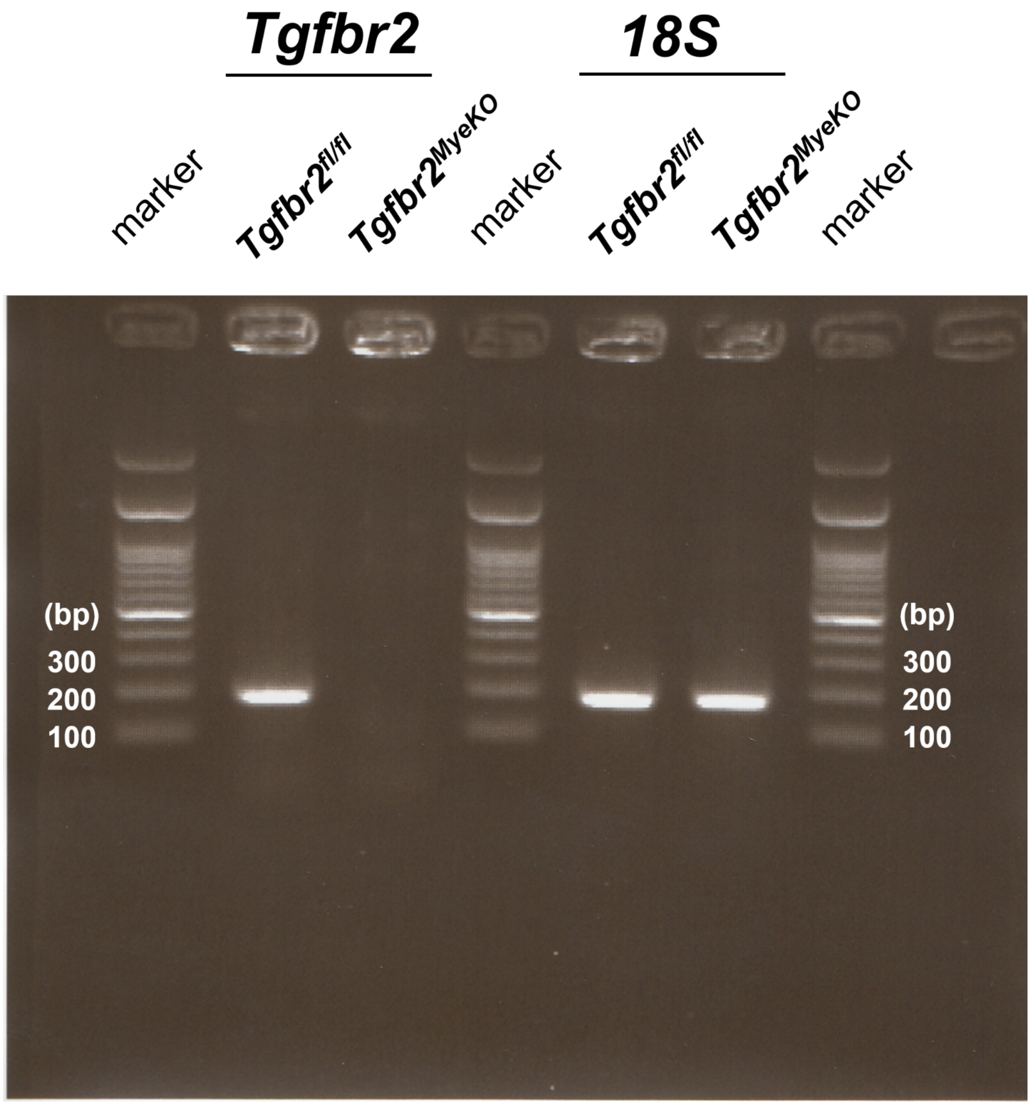

Products amplified by semi-quantitative reverse transcription polymerase chain reaction using *Tgfbr2* and *18s* primers were separated by 1.2% agarose gel electrophoresis.
